# Recent Progress in Human Placental Transcriptomics

**DOI:** 10.34763/devperiodmed.20192302.104108

**Published:** 2019-07-08

**Authors:** Marcin Jóźwik, Aleksandra Lipka

**Affiliations:** 1 Department of Gynecology and Obstetrics, School of Medicine, Collegium Medicum, University of Warmia and Mazury in Olsztyn, Olsztyn, Poland

**Keywords:** humans, placenta, pregnancy complications, transcriptome

## Abstract

The placenta serves as a metabolic, respiratory, excretory, and endocrine organ that provides appropriate conditions required for adequate feta/ development during pregnancy. The development of particular structures and proper functioning of the placenta are under the influence of sophisticated pathways controlled by the eexpression of substantial genes that are additionally regulated by long non-coding ribonucleic acids (RNAs). Disruptions to adaptive changes in the placenta/ transcriptome as a response to alterations in the feto-matema/ environment may be associated with pregnancy complications and compromised feta/ outcomes. The aim of the current paper was to present recent findings in transcriptomics of the human placenta. Different approaches in bioinformatic analyses of the RNA-sequencing results were presented. Novel knowledge about the genes and mechanisms that are crucial for the proper development of the placenta is essential for the understanding what stands behind both the norma/ and complicated pregnancy.

## Introduction

The placenta is essential for sustaining the growth of the fetus during gestation. Defects in the functioning of the organ can result in a vast array of serious disorders and conditions, including chronic and acute fetal hypoxia, deteriorated fetal well-being, intrauterine growth restriction (IUGR), or even fetal mortality. Consonant expression of crucial genes is required for the proper functioning of the placenta [1]. Spatiotemporal expression is a huge impediment in any transcriptome analysis, especially in the placenta, an organ that constantly adapts to feto-maternal environmental alterations. Nevertheless, ribonucleic acid *(RNA)-se*q*uencmg (RNAA-seq)*, also called whole transcriptome shotgun sequencing, has lately opened opportunities for the use of molecular resources and advanced transcriptomic analyses [2].

The aim of the current paper was to provide a brief overview of recent advances in the human placental transcriptome, together with the immediate implications of these findings. New technologies stand behind the new quality of results.

Recent studies using massive parallel sequencing techniques greatly contributed to the expansion of our knowledge on the placenta! transcriptional landscape in various eutherians: elephants, rats, pigs, beavers, and humans [3, 4, 5, 6]. Research specifically focused on global analysis of human placenta transcriptome using RNA-seq and bioinformatics tools to identify the profile of the gene expression and characterization of transcripts involved in the regulation of molecular mechanisms in late (36-41 weeks) single and twin uncomplicated human pregnancies, and important aspects of the placenta! functioning were introduced. Among the 228,044 identified transcripts, expression values were calculated for all the 38,948 transcriptional active regions (TARs). Within the TARs, there were 9,434 previously unknown regions that may play a specific role in the functioning of the placenta were identified and described.

**Fig. 1:**
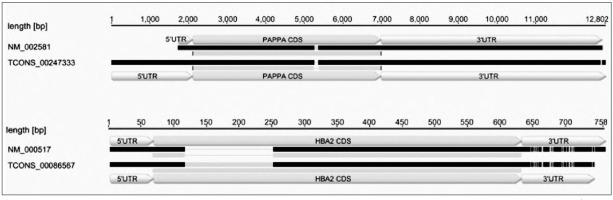
Pairwise alignment of examples showing differential splicing events identified in PAPPA and HBA2. Indication is made of the 5' untranslated region (UTR), coding DNA sequence (CDS), and 3' UTR. Adapted from [3].

Analyses revealed *HTRA1, EPAS1* and *PHLDA2* as the genes that showed the highest expression. It was reported that the plasma concentration of HTRA1 increases in pregnancies complicated by preeclampsia and IUGR [7], whilst EPAS1 is typed as a biomarker of early preeclampsia detection [8]. In contrast, PHLDA2 regulates fetal growth and placental development including placental lactogen production. Its increased placental expression is associated with reduced fetal growth and decreased birthweight of the newborn [9]. Increased PHLDA2 expression has also been correlated with a higher incidence of spontaneous abortions and fetal death [10]. Additionally, the observed association of PHLDA2 with pregnancies characterized by reduced fetal movements makes this gene a potential diagnostic marker of clinical significance [9]. Moreover, among 6,497 detected alternative splicing (AS) events, the most interesting ones in the context of placental research were those related to the *PAPPA* and *HBA* (Fig. 1).

The processes of AS generate a variety of transcripts and, hence, the formation of multiple isoforms of a single protein. This mechanism can substantially modulate the functions of genes influencing the development of various diseases [11]. The reduced level of the PAPPA complex is associated with abnormalities that may result in IUGR, premature delivery, miscarriage, and preeclampsia [12]. New transcripts and splicing forms provide new information on the expression profile in the human placenta during normal pregnancy, which may be investigated as potentially useful in clinical genomics.

Other studies concentrated on the thorough analyses of the placenta transcriptome, including those of long non-coding RNA (IncRNA), an important genomic element that modulates gene signaling pathways [5]. Analysis of the expression level revealed 5 IncRNA loci and 21 coding genes that were differentially expressed depending on the sex of the fetus. Of the IncRNAs, *HAND2-AS1* (on chromosome 4) and *XIST* (X chromosome) showed higher expression in female specimens. In contrast, *RP1-97J1.2* (chromosome 6), *AC010084.1* and *TTTY15* (Y chromosome) were expressed only in male specimens. In addition, in 37 genes, different expression was found at the level of exons or splicing sites. For example, a new splicing connection was identified in the genes *PSG4, ARHGAP45*, and *GATA2* engaged in the proper functioning of the endothelial barrier, embryogenesis, cytokinesis, differentiation and, therefore, having a significant impact on the course of pregnancy [5].

Changes were detected both in the exon expression and splicing sites in the *PPIG, HLA-DRB5, TOR1AIP1*, and *CSRNP1* genes. Changes in exon usage in various splicing forms were also found in IncRNAs, such as *H19* and *AC132217.4* (overexpression in female samples) as well as *AC005154.6* and *RP11-440I14.3* (overexpression in male samples). H19, a placenta-specific IncRNA, highly expressed during mammalian embryonic development [13], is implicated in the regulation of trophoblast proliferation, placental development, and fetal growth [14]. Moreover, the dynamic profile of H19 expression may support the normal course of pregnancy [15]. For the IncRNAs that were identified, a pool of 2,021 cis-target genes was also indicated. The functional analysis of target genes revealed 62 loci involved in embryonic development and 107 genes assigned to processes related to the development of blood vessels. Co-localization of the target gene and IncRNA may be very important in the regulation of molecular processes responsible for the proper functioning of the placenta and the course of pregnancy [16]. Investigation of the influence of lncRNA(s) on the expression of placental genes in late uncomplicated human pregnancy expands and updates our knowledge about placental functioning and constitutes the basis for further functional studies.

The results of the whole placental transcriptome sequencing established the foundations for the analyses of particular genes and enabled the identification of 1,364 bp of cDNA in human pregnancy-associated glycoprotein, also known as hPAG-L/pep [4]. *In silico* analysis indicated that cDNA encodes a 388 amino-acid sequence of hPAG-L polypeptide precursor. Two aspartic acid (D) residues specific for a catalytically active center (WFDTGSSNLWV^91-102^ and AIVDTGTSLLTG^274-285^) were predicted within the hPAG-L precursor, similarly as in other representatives of PAGs [17]. It was determined that 9,330 bp of the *hPAG-L* consists of nine exons and eight introns (A-H), a common feature among aspartic proteinases [18]. The Western heterologous analysis that was performed showed the presence of a dominant 60 kDa hPAG-L isoform among placental proteins. The hPAG-L proteins were localized in placental villi, syncytiotrophoblast cells, and in the vicinity of fetal blood vessels, which corresponds with PAG’s known expression features in different species [17]. These results further extend the current knowledge about the human genome and transcriptome, for the first time confirming the expression of the PAG-L family in the mature human placenta. It is essential for further functional studies to understand their role(s) during pregnancy.

Another approach in transcriptomics that was recently presented aimed to characterize the expression profile in placentas from pregnancies complicated by IUGR [6]. The use of restrictive bioinformatics algorithms allowed the identification of 37,501 TARs and the selection of 28 differentially expressed genes in IUGR samples. Functional analysis showed that most differentially expressed genes *(IL7R, PINLYP, FNDC4, ARMS2, LCK, ZAP70, BCL11B, SIRPG, ITK, BTNL9)* were involved in inflammatory processes and immunological disorders (Tab. I).

Additionally, changes that may be associated with the regulation of isoform expression and production of functional proteins were detected in *PSG6, SCEL, SIN3B, CTRP1, TFPI, PA28ß, PSAP, EVI5*, and *GPR126* genes that distorted expression and post-translational modifications seem to be crucial for the development of IUGR. Pregnancy-specific glycoproteins (PSGs) produced by placental syncytiotrophoblast affect the normal course of pregnancy [19]. The low level of PSG is associated with pregnancy complications, such as miscarriages, preeclampsia, or IUGR [20]. Moreover, *SIN3B* acts as a transcription factor regulating the expression of genes responsible for cell differentiation during embryonic development [21]. *S100A13* (S100 calcium-binding protein A13 gene) is a proangiogenic factor that regulates endothelial cell metabolism [22]. In addition, the higher exon skipping rate detected in the *GPR126* may be related to the dysfunction of umbilical cord endothelial cells and impaired placental angiogenesis [23]. Three different types of AS within *TFPI* can affect transcript stability resulting in the lack of functionality of the splice isoforms, leading to placental dysfunction that can be associated with the occurrence of vascular complications [24]. In addition, *TFPI* deficiency results in the mortality of embryos with growth retardation symptoms [25].

Alignment of RNA-seq reads to the human genome revealed 88,859 potential single nucleotide variant sites. Functional analysis of genes with detected nonsynonymous single nucleotide variant sites *(PIEZO1, PODXL, SWAP70)* showed their assignment to processes regulating cell adhesion via integrins. The expression of integrins determines the process of proper implantation and placentation, disorders in their expression lead to the development of diseases associated with vascular dysfunction [26]. PIEZO 1 is a molecular marker of placental flows and a therapeutic target in the treatment of vascular placental diseases [27]. The SWAP70 complex regulates the migration and invasion of trophoblastic cells during placental development [28], while the PODXL level in the blood is significantly elevated during preeclampsia and is a marker of endothelial cell dysfunction [29]. In summary, the above research indicates a wide range of possibilities of transcriptomic analyses for the diagnosis of pregnancy disorders.

**Table I:** Gene Ontology (GO) and the Kyoto Encyclopedia of Genes and Genomes (KEGG) analysis of differentially expressed genes identified in lUGR-affected placentas. Adapted from [6].

ID	Term	Gene
**G0:0050851**	antigen receptor-mediated signaling pathway	ITK, ZAP70, TESPA1, THEMIS, LCK
**G0:0050852**	T-cell receptor signaling pathway	ITK, ZAP70, TESPA1, THEMIS, LCK
**G0:0007159**	leukocyte cell-cell adhesion	SIRPG, ZAP70, TESPA1, IL7R, LCK
**GO:1903037**	regulation of leukocyte cell-cell adhesion	SIRPG, ZAP70, TESPA1, IL7R, LCK
**G0:0045321**	leukocyte activation	SIRPG, ITK, ZAP70, BCL11B, TESPA1, IL7R, THEMIS, LCK
**GO:0046649**	lymphocyte activation	SIRPG, ITK, ZAP70, BCL11B, TESPA1, IL7R, THEMIS, LCK
**G0:0042110**	T-cell activation	SIRPG, ITK, ZAP70, BCL11B, TESPA1, IL7R, THEMIS, LCK
**G0:0002520**	immune system development	ITK, ZAP70, BCL11B, TESPA1, IL7R, THEMIS, LCK, LTB
**GO:0048534**	hematopoietic or lymphoid organ development	ITK, ZAP70, BCL11B, TESPA1, IL7R, THEMIS, LCK, LTB
**G0:0030097**	hemopoiesis	ITK, ZAP70, BCL11B, TESPA1, IL7R, THEMIS, LCK,
**GO:0022409**	positive regulation of cell-cell adhesion	SIRPG, ZAP70, TESPA1, IL7R, LCK
**GO:1903039**	positive regulation of leukocyte cell-cell adhesion	SIRPG, ZAP70, TESPA1, IL7R, LCK
**G0:0050863**	regulation of T-cell activation	SIRPG, ZAP70, TESPA1, IL7R, LCK
**G0:0002521**	leukocyte differentiation	ITK, ZAP70, BCL11B, TESPA1, IL7R, THEMIS, LCK
**G0:0030098**	lymphocyte differentiation	ITK, ZAP70, BCL11B, TESPA1, IL7R, THEMIS, LCK
**G0:0030217**	T-cell differentiation	ITK, ZAP70, BCL11B, TESPA1, IL7R, THEMIS, LCK
**G0:0045058**	T-cell selection	ZAP70, BCL11B, THEMIS
**GO:0043368**	positive Cell selection	ZAP70, BCL11B, THEMIS
**G0:0033077**	T-cell differentiation in thymus	ZAP70, BCL11B, TESPA1, IL7R
**GO:0051251**	positive regulation of lymphocyte activation	SIRPG, ZAP70, TESPA1, IL7R, LCK
**G0:0050870**	positive regulation of T-cell activation	SIRPG, ZAP70, TESPA1, IL7R, LCK
**GO:0004715**	non-membrane spanning protein tyrosine kinase activity	ITK, ZAP70, LCK
**KEGG:04064**	NF-kappa B signaling pathway	ZAP70, LCK, LTB
**KEGG:04660**	T-cell receptor signaling pathway	ITK, ZAP70, LCK
**KEGG:05340**	Primary immunodeficiency	ZAP70, IL7R, LCK

## Concluding remarks

The recent progress in human placental transcriptomics provided data regarding the identification and global analysis of: transcriptome, expression of non-coding sequences, new gene variants and isoforms, and the mechanisms and effects of alternative splicing. The characterization of genes expressed in the placenta and their products may be the basis for the identification of molecular mechanisms determining effective reproduction. The following step in our understanding of distinctions between the normal and complicated pregnancy is to investigate the potentially crucial genes that were indicated together with mechanisms standing behind them *in vivo* in animal models. Additionally, such an approach may be further helpful in identifying marker genes and therapeutic targets. In the future, this new knowledge should have a direct impact on increasing the rate of successful pregnancies.

## List of Abbreviations

AS: alternative splicing
bp: base pairs
DNA: deoxyribonucleic acid
cDNA: complementary deoxyribonucleic acid
GO: Gene Ontology
hPAG-L/pep: human pregnancy-associated
glycoprotein: L / pepsinogen
IUGR: intrauterine growth restriction
kDa: kilodalton
KEGG: Kyoto Encyclopedia of Genes and Genomes
IncRNA: long non-coding RNA
PAG: pregnancy-associated glycoprotein
PSG: pregnancy-specific glycoprotein
RNA: ribonucleic acid
*RNA-seq –*: RNA-seq*uencing*
TAR: transcriptional active region
